# Technology-Facilitated Abuse in Relationships Scale: Psychometric Properties of Victimization and Perpetration Among Portuguese Adolescents

**DOI:** 10.1177/08862605251368832

**Published:** 2025-10-01

**Authors:** Maria Vale, Marlene Matos

**Affiliations:** 1 Psychology Research Centre (CIPsi), School of Psychology, University of Minho, Braga, Portugal

**Keywords:** adolescents, technology-facilitated dating abuse, victimization, perpetration, psychometric properties

## Abstract

Technology-facilitated dating abuse (TFDA) is a prevalent and harmful experience for adolescents. However, the absence of validated instruments in Portugal hinders early detection, prevention, and intervention. To address this gap, this study aims to: (a) analyze the psychometric properties of the Technology-Facilitated Abuse in Relationships (TAR) scale, recognized as one of the most reliable measures in this field; (b) estimate prevalence rates; and (c) examine associations with gender, age, self-esteem, health-related quality of life (HRQOL), and in-person dating violence. Between February and May 2023, 859 Portuguese adolescents (48.9% cisgender boys, 48.2% cisgender girls, and 2.9% gender-diverse; *M*_age_ = 15.27, *SD* = 1.68) enrolled in grades 7 to 12 across 25 schools in northern and central Portugal completed an online questionnaire. The TAR scale was translated and adapted following international guidelines. Confirmatory factor analysis supported the original four-factor structure—humiliation, monitoring/control, sexual coercion, and threats—for both victimization and perpetration, with adequate reliability, convergent, and discriminant validity. Prevalence rates showed that 11.9% reported victimization, 9.4% perpetration, and 34.5% victimization–perpetration. Monitoring/control behaviors were the most prevalent and bidirectional form. Gender and age differences emerged: Cisgender boys were more often uninvolved in monitoring/control, while cisgender girls were more frequently victims-perpetrators; adolescents aged 14 to 16 reported higher rates of victimization, particularly sexual coercion. TFDA was negatively correlated with self-esteem and HRQOL, and positively correlated with in-person dating victimization and perpetration. These results support the Portuguese version of the TAR scale as a valid instrument for research, clinical, and forensic use.

## Introduction

In Portugal, nearly 66% of adolescents report having a current or past dating relationship (União de Mulheres Alternativa e Resposta [Alternative and Response Women’s Union; UMAR], 2025; *N* = 6,732, aged 12–22). These relationships typically evolve from same-gender friendships to mixed-gender groups in early adolescence, followed by casual group-based dating in middle adolescence, and more stable, attached relationships in late adolescence (Connolly et al., 2014), playing a pivotal role in the development of identity, intimacy, and sexuality (Furman & Shaffer, 2003). Nowadays, in Western contexts, adolescent dating development unfolds amid rapid sociocultural shifts, including gender and sexual fluidity (e.g., Katz-Wise et al., 2023), a greater acceptance of diverse relationship types (e.g., Moreira et al., 2021), a heavy reliance on digital devices for initiating, maintaining, and ending these relationships (e.g., Baker & Carreño, 2016; Lenhart et al., 2015), and emerging risks such as technology-facilitated dating abuse (TFDA; e.g., Brown & Hegarty, 2018; Caridade & Braga, 2020; Rocha-Silva et al., 2021; Rodríguez-deArriba et al., 2021).

While terms and definitions continue to evolve (Brown & Hegarty, 2018; Rocha-Silva et al., 2021; Rodríguez-deArriba, 2021), TFDA broadly refers to a range of abusive and harmful technology-facilitated behaviors in relationships (Brown & Hegarty, 2021; Caridade & Braga, 2020; Vale et al., 2020). Rodríguez-deArriba et al. (2021) classify these behaviors into two macro-dimensions: nonsexual (e.g., verbal/emotional violence, relational violence, monitoring/control) and sexual (e.g., sexual pressure/coercion and forms of image-based sexual harassment/abuse).

Recent systematic reviews have identified 22 and 30 international TFDA measures (e.g., Brown & Hegarty, 2018; Martínez-Soto & Ibabe, 2022; Rodríguez-Domínguez et al., 2020). Based on COSMIN (COnsensus-based Standards for the selection of health Measurement INstruments) guidelines, the Cyber Dating Abuse Questionnaire (CDAQ), Abuse in Teen Relationships Questionnaire (CARPA), and Technology-Facilitated Abuse in Relationships (TAR) scale are highly recommended for their robust methodological and psychometric properties (Martínez-Soto & Ibabe, 2022). While the CDAQ (Borrajo et al., 2015) has been validated in Portugal (Caridade & Braga, 2019) and other countries, it focuses on adults aged 18 to 30. CARPA, designed for Spanish adolescents aged 11 to 18, assesses in-person and online dating violence, but focuses only on online direct aggression and control (Calvete et al., 2021). In contrast, the TAR scale, administered to Australian youth aged 16 to 24 (Brown & Hegarty, 2021), is particularly suitable for adaptation in Portugal, where current assessments often rely on purpose-built items for specific studies (e.g., UMAR, 2025) and/or on a limited seven-item subscale of the Portuguese version of the Conflict in Adolescent Dating Relationships Inventory (CADRI-P; Lucas et al., 2017), which primarily captures insults and monitoring/control behaviors during an argument.

The TAR stands out for several reasons: (a) It was developed using a theory-driven approach and ensured face and content validity through expert consultations, youth discussion groups, and questionnaires; (b) it adopts a holistic definition of key constructs, encompassing various modern technologies—from everyday devices to sophisticated applications—while recognizing the evolving dynamics of adolescents’ dating relationships—whether monogamous or open, straight or gay, casual or serious, short-term or long-term; (c) it addresses multiple forms of abuse, including humiliation, monitoring/control, sexual coercion, and threats (Brown & Hegarty, 2021)—and notably includes a sexual form, filling a gap identified in recent reviews where only 6 of 20 multidimensional behavioral sets considered this dimension (Rocha-Silva et al., 2021), despite its documented prevalence (e.g., Ellyson et al., 2021; Reed et al., 2017; Stonard, 2021; Zweig et al., 2013) and association with distressed responses (e.g., Reed et al., 2017); (d) it acknowledges the bidirectional nature of the phenomenon, challenging traditional paradigms in victimology/criminology that dichotomize individuals as victims or perpetrators (Jennings et al., 2012); (e) finally, its development included exploratory factor analysis, which identified the factor structure of victimization, explaining 53.4% of the total variance, with strong internal consistency (Cronbach’s alphas: .80–.88; Brown & Hegarty, 2021).

Studies in the United States and Europe show varying TFDA involvement rates: noninvolvement (23.9%–27.1%), victimization (17.6%–24.7%), perpetration (0.7%–6.3%), and victimization–perpetration (8.6%–48.9%; Ellyson et al., 2021, *N* = 696, aged 16–22; Stonard, 2021, *N* = 277, aged 12–18; Zweig et al., 2013, *N* = 3,745, grades 7–12). Nonsexual forms–particularly related to monitoring/control—are most common (e.g., Ellyson et al., 2021; Reed et al., 2017; Stonard, 2021) and bidirectional (e.g., Ellyson et al., 2021). Specifically in Portugal, the most recent national data on dating violence indicated that 19.8% of participants reported victimization through social networking sites (*N* = 4,493, aged 12–22; UMAR, 2025), and that support was provided to 66 victims of dating violence up to the age of 17, although the type of violence was not specified (Associação Portuguesa de Apoio à Vítima [Portuguese Victim Support Association], 2025; *N* = 1,023 victims, ages up to 17–65+).

Gender differences in TFDA are mixed: While nonsexual behaviors show lack consistent patterns, sexual forms typically involve girls as victims and boys as perpetrators (e.g., Ellyson et al., 2021; Reed et al., 2017; Stonard, 2021; Zweig et al., 2013). The average age of onset for most TFDA behaviors was 16, with some adolescents reporting experiences as early as age 11 (e.g., Ellyson et al., 2021). Intrapersonal and interpersonal factors have been linked to TFDA (Caridade & Braga, 2020), including low self-esteem (Smith et al., 2018), reduced health-related quality of life (HRQoL; Ortega-Barón et al., 2022), and a significant overlap with in-person dating violence (Rodríguez-deArriba et al., 2024). The consequences of TFDA are far-reaching, encompassing emotional and mental health-related difficulties (e.g., low self-esteem, depressive mood, emotional overwhelm, psychological distress), risky behaviors (e.g., substance use), self-restricting behaviors (e.g., social isolation), and self-harming behaviors (e.g., suicide attempts; Afrouz & Vassos, 2024; Hellevik, 2019; Torp Løkkeberg et al., 2024).

### The Current Study

To our knowledge, this is the first study to test the TAR scale’s proposed factorial structure for victimization, extend its validation to perpetration, and address the lack of instruments measuring TFDA among Portuguese adolescents while estimating prevalence rates and identifying correlates. These efforts enable cross-country comparative studies and contribute to early detection, prevention, and intervention efforts. This study addressed four research questions: (a) Is the TAR scale valid and reliable for assessing TFDA among Portuguese adolescents? (b) To what extent are adolescents involved in TFDA as victims, perpetrators, and victims-perpetrators? (c) Are there gender and age differences in TFDA roles? (d) How does TFDA relate to opposing constructs, such as adolescents’ self-esteem and HRQoL, and similar constructs, such as in-person dating violence? It is hypothesized that the TAR scale will confirm its original four-factor structure with good psychometric properties (H1); that TFDA will be prevalent, with a substantial proportion of adolescents involved as victims-perpetrators (H2a); that monitoring/control will be the most common and bidirectional form (H2b); that cisgender girls will more often be victims and cisgender boys perpetrators of sexual forms (H3a); that middle to late adolescents—defined here as those aged 14 to 16 (middle) and 17 to 18 (late)—will be more frequently involved in both victimization and perpetration of sexual and nonsexual forms (H3b); and that TFDA will negatively correlate with self-esteem (H4a) and HRQoL (H4b), and positively with in-person dating victimization and perpetration (H4c).

## Method

### Translation of TAR Scale

Following the International Test Commission Guidelines for Translating and Adapting Tests (International Test Commission, 2017), this process included: (a) authorization from the original authors; (b) forward translation by two bilingual researchers, reconciled by a third; (c) back-translation by a bilingual researcher and a native English speaker, reconciled by a third bilingual researcher; (d) clarification of terms (e.g., “nudes”) and alignment of item phrasing with Portuguese dating violence instruments by preceding items with “He/She” for victimization and “I” for perpetration (e.g., Saavedra et al., 2011); (e) expert review by nine psychology professionals; (f) a pilot study was conducted with to evaluate item clarity and comprehension. The instrument was reviewed by 10 Portuguese adolescents, selected through convenience sampling, with proportional representation of cisgender boys and girls aged 12 to 18 and enrolled in grades 7 to 12. The characteristics of this pilot sample were aligned with those of the main validation sample in terms of gender, age, nationality, and school grade. No modifications to the scale were necessary based on their feedback. These participants were excluded from the primary analysis; and (g) final approval was obtained from the original authors.

### Procedures

The study was approved by the Ethics Committee for Research in Social and Human Sciences of the University of Minho (CEICSH-109/2021), the Data Protection Department of the University of Minho (DPO-REG-ID-2020-0000; DPO-AIPD-ID-2020-0000), the Portuguese General Directorate of Education (DGE-079070000), and the executive boards of participating schools. A descriptive, observational, cross-sectional design was used. Using the DGE registry, we selected public and private schools offering regular and vocational education (grades 7–12) by NUTS 2 region, with a focus on the North and Center regions—where our research team is based—and sought to include schools in both urban and rural areas. This geographic focus ensured: (a) streamlined ethics authorization; (b) the possibility of on-site monitoring of data collection fidelity; and (c) containment of study costs and timelines. Formal invitations were emailed to school directors. Twenty-five schools agreed to participate and received a letter detailing the study procedures, ethical considerations, and consent forms requiring signatures from adolescents and parents. All students present on the prescheduled data collection day who returned signed forms were eligible, constituting a nonprobabilistic convenience sample. Data collection occurred from February to May 2023 within a single school day. Adolescents provided digital consent and were reminded not to include their names, of the option to skip questions, and of the freedom to withdraw at any time. They completed a 45-min online questionnaire on Qualtrics, using PCs, tablets, or smartphones, in classrooms or libraries, with the presence of a teacher, lead researcher, and, when necessary, a master’s or PhD psychology student. No financial compensation was provided. However, following data collection, adolescents attended a 45-min awareness session on TFDA, covering the phenomenon, safety tips, and support contacts, including the project team, the APAV, and the Psychology Association of the University of Minho (APsi). The project’s email was also provided for questions, assistance, or accessing results.

### Participants

Participants were eligible if they were adolescents aged 12 to 18, of Portuguese nationality, had dated in the past 12 months, and had no cognitive or severe learning difficulties (as assessed by teachers). Of 1,855 adolescents, 996 were excluded: 14 with incomplete dating status, 848 who had not dated in the past 12 months,^1^ 84 without Portuguese nationality, 28 with no responses on the TAR scale, and 22 identified as outliers. As summarized in Table 1, most adolescents were cisgender boys (48.9%), aged 14 to 16 (57%), White (93.2%), heterosexual (84.9%), and had been in a committed relationship within the last 12 months (47.7%). Digital devices played a key role in their relationships: 41.7% spent over 5 hr of daily communicating, 57.6% used them to initiate relationships, 76.8% to share personal information, 38.2% for sexual intimacy, 79.4% for conflict resolution, 53% for breakups, and 73.9% for reconciliation. Furthermore, 67.3% attended public schools, 44.9% were in grades 9 to 10, and 51.3% lived in urban areas.

**Table 1.** Description of the Eligible Sample (*N* = 859).

Variables	*n* (%)
Gender	
Cisgender boys	420 (48.9)
Cisgender girls	414 (48.2)
Binary and nonbinary transgender	15 (1.8)
Gender questioning	10 (1.1)
Age group (years)	
12–13	144 (16.8)
14–16	490 (57)
17–18	225 (26.2)
Ethnicity	
White	801 (93.2)
Black	36 (4.2)
Roma/Romani	16 (1.9)
Asian	1 (0.1)
Multiethnic	5 (0.6)
Sexual orientation	
Heterosexual	729 (84.9)
Bisexual	84 (9.8)
Gay/lesbian	29 (3.4)
Other	16 (1.9)
Relationship status in the last 12 months	
Committed dating	410 (47.7)
Casual dating	368 (42.8)
Committed and casual dating	81 (9.4)
Use of digital devices in the dating relationship	
Daily digital communication with their partner (hrs)	
1–2	251 (29.2)
3–4	242 (28.2)
More than 5	358 (41.7)
Initiate the relationship	495 (57.6)
Share personal information, interests, and experiences	660 (76.8)
Engage in sexual intimacy (i.e., sexting)	328 (38.2)
Discuss and resolve conflicts	682 (79.4)
Communicate breakups	455 (53)
Reconcile after an argument and/or rekindle the relationship	635 (73.9)
Type of school	
Public	578 (67.3)
Private/professional	281 (32.7)
Education level	
7th–8th grades	226 (26.3)
9th–10th grades	386 (44.9)
11th–12th grades	247 (28.8)
Residential zone	
Urban	441 (51.3)
Rural	414 (48.2)

*Note*. Missing data ranged from 0.1% for sexual orientation, 0.5% for residential zone, and up to 0.9% for daily digital communication with their partner.

### Measures

#### Sociodemographic Questionnaire

Single items were developed to collect data on gender,^2^ age, ethnicity, sexual orientation, relationship status, use of digital devices in dating relationships, type of school, education level, and residential zone.

#### Technology-Facilitated Abuse in Relationships Scale

These pre-survey definitions were provided for adolescents who had dated in the past 12 months (Brown & Hegarty, 2021):

a. Dating relationships. We define dating as two or more people in an emotionally and/or physically intimate relationship. The relationship may or may not be sexual. It may be casual or serious, short-term or long-term, straight, gay, monogamous, or open. Terms used to describe these kinds of relationships include boyfriend, girlfriend, going out, hanging out, hooking up, seeing each other, friends with benefits, and unofficially dating. In this survey, we will use the terms girlfriend and boyfriend to refer to dating relationships.
b. Digital devices. A digital device includes any type of modern-day technology used to interact with other people. Examples include (but are not limited to) mobile phones, smartphones, computers, tablets, laptops, notepads, the internet, social media, GPS devices, software, and apps.

The TAR scale (Brown & Hegarty, 2021) is a 30-item paired self-report measure designed to assess both victimization and perpetration of TFDA over the past 12 months across four dimensions: humiliation (10 items, e.g., “He/she shared a hurtful meme about me on a digital device” [victimization, α = .81], “I shared a hurtful meme about him/her on a digital device” [perpetration, α = .77]); monitoring/control (7 items, e.g., “He/she checked to see who I was communicating with on my digital device in a way that made me feel uncomfortable” [victimization, α = .72], “I checked to see who he/she was communicating with on his/her digital device” [perpetration, α = .72]); sexual coercion (8 items, e.g., “He/she pressured me to send intimate/sexual image(s) (nudes) of myself” [victimization, α = .77], “I pressured him/her to send intimate/sexual image(s) (nudes) of him/herself” [perpetration, α = .71]; and threats (5 items, e.g., “He/she threatened on a digital device to emotionally hurt me” [victimization, α = .66], “I threatened on a digital device to emotionally hurt him/her” [perpetration, α = .63]). Items were initially rated on a 6-point Likert scale (0 = *never* to 5 = *daily/almost daily*). However, responses were recoded into a 4-point scale (0 = *never* to 3 = *monthly, weekly, daily/almost daily*) to address floor and ceiling effects. This approach allows for the retention of information on the occurrence and frequency of behaviors, providing greater sensitivity to repeated or escalating patterns of TFDA, which may be overlooked if a binary scoring format had been used. Higher scores correspond to a higher prevalence of victimization (α = .82) and/or perpetration (α = .82).

#### Rosenberg Self-Esteem Scale

The Rosenberg Self-Esteem Scale (RSES; Rosenberg, 1965; Portuguese Adolescent Validation of Santos & Maia, 2003) is a 10-item self-report scale that evaluates general feelings of self-esteem (e.g., “On the whole, I am satisfied with myself.”). Responses are rated on a 4-point Likert scale (1 = *strongly disagree* to 4 = *strongly agree*). Higher scores indicate a higher level of self-esteem (α = .86).

#### Kidscreen-27

The Kidscreen-27 (Ravens-Sieberer et al., 2007; Portuguese Adolescent Validation of Gaspar & Matos, 2008) is a 27-item self-report scale that evaluates HRQoL over the past week across five dimensions: physical well-being (5 items, e.g., “Have you physically felt fit and well?”; α = .60), psychological well-being (7 items, e.g., “Has your life been enjoyable?”; α = .89), autonomy and parent relations (7 items, e.g., “Have you had enough time for yourself?”; α = .88), peers and social support (4 items, e.g., “Have you spent time with your friends?”; α = .91), and the school environment (4 items, e.g., “Have you been happy at school?”; α = .77). Responses are rated on a 5-point Likert scale (1 = *never/nothing* to 5 = *always/totally*). The Rasch person parameter replaces sum scores and is then translated into *T*-values. Higher scores correspond to a better perception of HRQoL in the respective dimension (α = .92).

#### Conflict in Adolescent Dating Relationships Inventory

The CADRI (Wolfe et al., 2001; Portuguese Adolescent Validation of Saavedra et al., 2011) is a 25-paired item self-report scale that assesses the prevalence of victimization and perpetration over the past 12 months across 5 dimensions: physical abuse (4 items, e.g., “He/she threw something at me” [victimization, α = .45], “I threw something at him/her” [perpetration, α = .30]); verbal/emotional abuse (10 items, e.g., “He/she said things just to make me angry” [victimization, α = .85], “I said things just to make him/her angry” [perpetration, α = .80]); relational abuse (3 items, e.g., “He/she tried to turn my friends against me” [victimization, α = .82], “I tried to turn his/her friends against him/her” [perpetration, α = .55]); sexual abuse (4 items, e.g., “He/she touched me sexually when I didn’t want him/her to” [victimization, α = .63], “I touched her/him sexually when she/he didn’t want me to” [perpetration, α = .58]); and threatening behaviors (4 items, e.g., “He/she threatened to hurt me” [victimization, α = .42], “I threatened to hurt him/her” [perpetration, α = .19]). Responses are rated on a 4-point scale (0 = *never* to 3 = *always*). Higher scores correspond to a higher prevalence of victimization (α = .88) and/or perpetration (α = .82). The CADRI was administered only to adolescents who reported being in a committed relationship within the past 12 months, which aligns with recommendations from the Portuguese validation of the scale (e.g., Saavedra et al., 2011). Of the 491 adolescents who met this condition (see Table 1), 426 provided valid responses to the CADRI and were therefore included in the analyses (see Table 7).

### Data Analysis

For preliminary analyses, Little’s missing completely at random (MCAR) test (Little, 1988) indicated insufficient evidence to reject the assumption that data for the TAR scale, RSES, KIDSCREEN-27, and CADRI were missing completely at random (χ²[4220] = 3463.06, *p* > .05). Approximately 4% of cases had less than 10% missing data (0.7%–2.7%), which were imputed using multivariate imputation by chained equations (MICE; van Buuren & Groothuis-Oudshoorn, 2011) under the random forest algorithm (Shah et al., 2014), with 10 multiple imputations and a maximum of 50 iterations. Density plots confirmed similar distributions between the original and imputed data. Outliers were identified using Mahalanobis distance. Item analysis included descriptive statistics and normality tests (Kolmogorov–Smirnov, Shapiro–Wilk, skewness [*sk*] >3, kurtosis [*ku*] >7; Finney & DiStefano, 2013). To address RQ1, confirmatory factor analysis (CFAs) tested the original four-factor structure using the MLM estimator, with residual variances fixed to 1 and allowed to correlate. Adequate fit criteria included chi-square/degrees of freedom (χ^2^/*df*) ≤5, comparative fit index (CFI) and Tucker–Lewis index (TLI) ≥0.90, root mean square error of approximation (RMSEA) ≤0.08 with a nonsignificant *p*-close (*p* ≥ .05), standardized root mean square residual (SRMR) ≤0.08 (Marôco, 2021), and factor loadings ≥ 0.32 (Tabachnick & Fidell, 2007). Model re-specifications adhered to modification indices aligned with theoretical considerations (Hair et al., 2019). Reliability was evaluated using Cronbach’s alpha ≥.70 (Dunn et al., 2014), mean inter-item correlations (MIIC) between .15 and .50 (Clark & Watson, 1995), and corrected item-total correlations (CITC) ≥.20 (Nunnally & Bernstein, 1994). Convergent validity was assessed using average variance extracted (AVE) ≥0.50 (Fornell & Larcker, 1981) and composite reliability (CR) ≥ 0.60 (Hair et al., 2019), while discriminant validity was evaluated using the heterotrait–monotrait ratio of correlations (HTMT) ≤.85 (Henseler et al., 2015). To address RQ2, descriptive statistics were used to estimate the prevalence of adolescents’ involvement in TFDA. For RQ3, chi-square or Fisher’s exact tests (when >20% of expected cell counts were below five; Field, 2017) examined associations with gender and age, with adjusted standardized residuals used to identify significant deviations from expected values (*z* ≥ 1.96, *z* ≤ −1.96). For RQ4, Spearman correlations assessed the associations with self-esteem, HRQoL, and in-person dating violence. All effect sizes were interpreted as small (*rs* ≃ .10), moderate (*rs ≃* .30), and large (*rs ≃* .50; Cohen, 1988). Statistical significance was determined using a conventional alpha level of *p* < .05. R (R Core Team, 2023) was used for missing data imputation, CFAs, and validity assessments, using MICE (van Buuren & Groothuis-Oudshoorn, 2011), VIM (Kowarik & Templ, 2016), lavaan (Rosseel, 2012), and semTools (Jorgensen et al., 2025) packages. SPSS 29 (IBM Corp., 2022) was employed for all other analyses.

## Results

### Psychometric Properties

The results support our first hypothesis. As shown in Tables 2 and 3, the most frequently reported behavior was item 15 on the corresponding items of the victimization and perpetration scales: “He/she checked to see who I was communicating with on my digital device in a way that made me feel uncomfortable” (18.3% victimization) and “I checked to see who he/she was communicating with on his/her digital device” (19.5% perpetration). The least frequent behaviors were “He/she signed me onto a pornography site without my permission” (item 1, 1.5% victimization) and “I pressured him/her to engage in phone sex” (item 20, 1% perpetration). Normality tests indicated highly positively skewed and leptokurtic distributions, consistent with research on violence-related topics.

**Table 2.** Descriptive and Item Analyses of the Victimization Scale (*N* = 859).

Item	Frequency Distribution, *n* (%)				*M*	*SD*	Sk (SE = 0.08)	Ku (SE = 0.17)
	Never	Once	Few Times	Monthly, Weekly, Daily/Almost Daily				
1. Signed me up on a pornography site without my permission.	846 (98.5)	5 (0.6)	3 (0.3)	5 (0.6)	0.03	0.27	9.71	98.10
2. Started a social networking page for posting negative information about me.	833 (97)	21 (2.4)	4 (0.5)	1 (0.1)	0.04	0.23	7.34	63.46
3. Threatened to distribute intimate/sexual image(s) (nudes) of me.	837 (97.4)	16 (1.9)	4 (0.5)	2 (0.2)	0.03	0.24	8.30	79.24
4. Told me on a digital device to harm myself.	828 (96.4)	21 (2.4)	7 (0.8)	3 (0.3)	0.05	0.29	6.79	51.77
5. Edited a photo or video of me in an offensive manner and sent it to me.	836 (97.3)	17 (2)	5 (0.6)	1 (0.1)	0.03	0.23	7.72	68.22
6. Threatened on a digital device to physically hurt me.	832 (96.9)	18 (2.1)	8 (0.9)	1 (0.1)	0.04	0.26	6.85	51.75
7. Changed an aspect of my online profile without my permission.	838 (97.6)	15 (1.7)	5 (0.6)	1 (0.1)	0.03	0.22	8.07	74.05
8. Posted something negative through my account without my permission.	837 (97.4)	13 (1.5)	7 (0.8)	2 (0.2)	0.04	0.26	7.78	66.62
9. Threatened on a digital device to physically hurt my family or friends.	837 (97.4)	16 (1.9)	5 (0.6)	1 (0.1)	0.03	0.23	7.89	71.05
10. Shared a hurtful meme about me on a digital device.	828 (96.4)	21 (2.4)	8 (0.9)	2 (0.2)	0.05	0.28	6.61	48.80
11. Made me remove or add contact(s) on my digital device.	773 (90)	54 (6.3)	26 (3)	6 (0.7)	0.14	0.48	3.66	13.88
12. Made me stop interacting with another person(s) on my digital device.	735 (85.6)	70 (8.1)	43 (5)	11 (1.3)	0.22	0.59	2.86	7.75
13. Pressured me to share my password(s) with him/her.	831 (96.7)	14 (1.6)	10 (1.2)	4 (0.5)	0.05	0.32	6.73	48.44
14. Made me disclose digital conversation(s) I have had with another person(s) to him/her.	789 (91.9)	38 (4.4)	29 (3.4)	3 (0.3)	0.12	0.44	3.87	14.99
15. Checked to see who I was communicating with on my digital device in a way that made me feel uncomfortable.	702 (81.7)	66 (7.7)	78 (9.1)	13 (1.5)	0.30	0.70	2.21	3.76
16. Logged onto my digital device without my permission.	790 (92)	33 (3.8)	30 (3.5)	6 (0.7)	0.13	0.47	3.94	15.48
17. Monitored where I am via tracking software.	814 (94.8)	18 (2.1)	17 (2)	10 (1.2)	0.10	0.44	5.05	25.94
18. Pressured me to send intimate/sexual image(s) (nudes) of myself.	818 (95.2)	22 (2.6)	15 (1.7)	4 (0.5)	0.07	0.36	5.47	31.72
19. Pressured me on a digital device to send sexually explicit messages.	842 (98)	11 (1.3)	4 (0.5)	2 (0.2)	0.03	0.23	9.28	96.59
20. Pressured me to engage in phone sex.	840 (97.8)	13 (1.5)	5 (0.6)	1 (0.1)	0.03	0.22	8.46	80.69
21. Pressured me on a digital device to engage in sexual acts.	844 (98.3)	8 (0.9)	7 (0.8)	0 (0)	0.03	0.20	8.53	75.14
22. Pressured me on a digital device to discuss sexual issues.	834 (97.1)	14 (1.6)	11 (1.3)	0 (0)	0.04	0.26	6.56	43.97
23. Pressured me to engage in sexual activity via live video.	845 (98.4)	10 (1.2)	3 (0.3)	1 (0.1)	0.02	0.19	10.24	120.52
24. Sent me unwelcome intimate/sexual image(s) (nudes).	823 (95.8)	16 (1.9)	19 (2.2)	1 (0.1)	0.07	0.34	5.37	29.16
25. Shared intimate/sexual images (nudes) or videos of me without my permission.	839 (97.7)	19 (2.2)	1 (0.1)	0 (0)	0.02	0.16	6.98	52.42
26. Sent me threatening messages on a digital device.	832 (96.9)	22 (2.6)	4 (0.5)	1 (0.1)	0.04	0.23	7.19	61.01
27. Threatened on a digital device to emotionally hurt me.	815 (94.9)	28 (3.3)	14 (1.6)	2 (0.2)	0.07	0.34	5.26	29.79
28. Threatened on a digital device to damage things that are important to me.	842 (98)	14 (1.6)	3 (0.3)	0 (0)	0.02	0.17	8.24	74.57
29. Made me feel threatened if I ignored their calls or messages.	816 (95)	23 (2.7)	20 (2.3)	0 (0)	0.07	0.34	4.85	23.11
30. Threatened on a digital device to physically hurt him/herself if I didn’t do what he/she wanted.	825 (96)	26 (3)	6 (0.7)	2 (0.2)	0.05	0.28	6.49	48.73

*Note. M* = mean; *SD* = standard deviation; Sk = skewness; Ku = kurtosis; SE = error.

**Table 3.** Descriptive and Item Analyses of the Perpetration Scale (*N* = 859).

Item	Frequency Distribution, *n* (%)				*M*	*SD*	Sk (SE = 0.08)	Ku (SE = 0.17)
	Never	Once	Few Times	Monthly, Weekly, Daily/Almost Daily				
1. Signed him/her onto a pornography site without his/her permission.	842 (98)	8 (0.9)	4 (0.5)	5 (0.6)	0.04	0.28	8.80	81.80
2. Started a social networking page for posting negative information about him/her.	845 (98.4)	11 (1.3)	2 (0.2)	1 (0.1)	0.02	0.18	10.57	132.36
3. Threatened to distribute intimate/sexual image(s) (nudes) of him/her.	844 (98.3)	12 (1.4)	2 (0.2)	1 (0.1)	0.02	0.18	10.20	124.16
4. Told him/her on a digital device to harm him/her.	845 (98.4)	9 (1)	4 (0.5)	1 (0.1)	0.02	0.20	9.93	110.53
5. Edited a photo or video of him/her in an offensive manner and sent it to him/her.	837 (97.4)	14 (1.6)	6 (0.7)	2 (0.2)	0.04	0.25	7.95	70.39
6. Threatened on a digital device to physically hurt him/her.	838 (97.6)	15 (1.7)	6 (0.7)	0 (0)	0.03	0.21	7.40	58.32
7. Changed an aspect of his/her online profile without his/her permission.	842 (98)	14 (1.6)	3 (0.3)	0 (0)	0.02	0.17	8.24	74.57
8. Posted something negative through his/her account without his/her permission.	842 (98)	15 (1.7)	2 (0.2)	0 (0)	0.02	0.16	8.07	72.11
9. Threatened on a digital device to physically hurt his/her family or friends.	839 (97.7)	16 (1.9)	4 (0.5)	0 (0)	0.03	0.19	7.59	62.82
10. Shared a hurtful meme about him/her on a digital device.	816 (95)	30 (3.5)	11 (1.3)	2 (0.2)	0.07	0.32	5.47	33.07
11. Made him/her remove or add contact(s) on his/her digital device.	786 (91.5)	47 (5.5)	21 (2.4)	5 (0.6)	0.12	0.44	4.06	17.49
12. Made him/her stop interacting with another person(s) on his/her digital device.	781 (90.9)	49 (5.7)	24 (2.8)	5 (0.6)	0.13	0.45	3.86	15.54
13. Pressured him/her to share his/her password(s) with me.	829 (96.5)	20 (2.3)	7 (0.8)	3 (0.3)	0.05	0.29	6.89	53.11
14. Made him/her disclose digital conversation(s) he/she has had with another person(s) to me.	804 (93.6)	31 (3.6)	22 (2.6)	2 (0.2)	0.09	0.39	4.46	20.41
15. Checked to see who he/she was communicating with on his/her digital device.	692 (80.6)	65 (7.6)	91 (10.6)	11 (1.3)	0.33	0.71	2.04	2.87
16. Logged onto his/her digital device without his/her permission.	801 (93.2)	38 (4.4)	16 (1.9)	4 (0.5)	0.10	0.39	4.67	23.54
17. Monitored where he/she was via tracking software.	810 (94.3)	25 (2.9)	17 (2)	7 (0.8)	0.09	0.42	4.99	26.02
18. Pressured him/her to send intimate/sexual image(s) (nudes) of him/her.	838 (97.6)	15 (1.7)	3 (0.3)	3 (0.3)	0.03	0.25	8.72	86.24
19. Pressured him/her on a digital device to send sexually explicit messages.	849 (98.8)	9 (1)	0 (0)	1 (0.1)	0.01	0.14	13.74	236.12
20. Pressured him/her to engage in phone sex.	850 (99)	6 (0.7)	2 (0.2)	1 (0.1)	0.02	0.16	12.93	189.85
21. Pressured him/her on a digital device to engage in sexual acts.	846 (98.5)	10 (1.2)	3 (0.3)	0 (0)	0.02	0.16	9.52	98.83
22. Pressured him/her on a digital device to discuss sexual issues.	842 (98)	13 (1.5)	4 (0.5)	0 (0)	0.02	0.18	8.28	74.40
23. Pressured him/her to engage in sexual activity via live video.	849 (98.8)	8 (0.9)	1 (0.1)	1 (0.1)	0.02	0.16	13.01	201.48
24. Sent him/her unwelcome intimate/sexual image(s) (nudes).	825 (96)	21 (2.4)	12 (1.4)	1 (0.1)	0.06	0.30	5.89	37.09
25. Shared intimate/sexual images (nudes) or videos of him/her without his/her permission.	849 (98.8)	8 (0.9)	1 (0.1)	1 (0.1)	0.02	0.16	13.01	201.48
26. Sent him/her threatening messages on a digital device.	842 (98)	14 (1.6)	0 (0)	3 (0.3)	0.03	0.22	10.50	127.85
27. Threatened on a digital device to emotionally hurt him/her.	842 (98)	13 (1.5)	2 (0.2)	2 (0.2)	0.03	0.21	9.87	113.27
28. Threatened on a digital device to damage things that are important to him/her.	846 (98.5)	12 (1.4)	1 (0.1)	0 (0)	0.02	0.14	9.01	89.81
29. Threatened him/her if he/she ignored my calls or messages.	839 (97.7)	14 (1.6)	6 (0.7)	0 (0)	0.03	0.21	7.58	61.07
30. Threatened on a digital device to physically hurt myself if he/she didn’t do what I wanted.	848 (98.7)	11 (1.3)	0 (0)	0 (0)	0.01	0.11	8.68	73.54

*Note. M* = mean; *SD* = standard deviation; Sk = skewness; Ku = kurtosis; SE = standard error.

As presented in Table 4, the victimization scale exhibited good fit (S-Bχ²/*df* = 1.14, CFI = 0.94, TLI = 0.94, RMSEA = 0.03, *p*-close >.05, and SRMR = 0.07). Factor loadings were adequate, ranging from 0.32 to 0.77, except for item 25: “Shared intimate/sexual images (nudes) or videos of me without my permission.” The perpetration scale initially exhibited suboptimal fit, which improved significantly after adding theoretically justified error term correlations (S-Bχ²/*df* = 1.13, CFI = 0.91, TLI = 0.90, RMSEA (90%) = 0.03, *p*-close >.05, and SRMR = 0.07). Factor loadings were adequate, ranging from 0.35 to 0.91, except for item 24: “Sent him/her unwelcome intimate/sexual image(s) (nudes)” and item 28: “Threatened on a digital device to damage things that are important to him/her.” The detailed CFA models with standardized loadings and error terms are available in Supplemental Figure 1.

**Table 4.** Model Fit Statistics for Confirmatory Factor Analysis (*N* = 859).

Model	S-Bχ^2^	*df*	S-Bχ^2^/*df*	CFI	TLI	RMSEA	90% CI	*p*-close	SRMR
Victimization scale	454.193*	399	1.14	0.94	0.94	0.03	[0.01, 0.04]	>.05	0.07
Perpetration scale									
Model without correlated error terms	509.108	399	1.28	0.77	0.75	0.04	[0.03, 0.05]	>.05	0.08
Model with correlated error terms	439.077*	389	1.13	0.91	0.90	0.03	[0.01, 0.04]	>.05	0.07

*Note.* S-Bχ^2^ = Satorra-Bentler chi-square test; *df* = degrees of freedom; CFI = comparative fit index; TLI = Tucker-Lewis index; RMSEA = root mean square error of approximation (90% confidence interval); SRMR = standardized root mean square residual; CI = confidence interval.

*p< .05.

As outlined in Table 5, both scales showed good internal consistency (victimization: 0.66–0.82, perpetration: 0.63–0.82) and homogeneity (victimization: 0.16–0.31, perpetration: 0.17–0.30). Item discriminative power was acceptable, with only item 25 (victimization) and items 24 and 28 (perpetration) falling below the CITC threshold of 0.20 (victimization: 0.18–0.66, perpetration: 0.08–0.71). Furthermore, both scales exhibited small to moderate positive correlations (victimization: 0.19–0.35; perpetration: 0.19–0.26). While AVE values fell below the 0.50 threshold (victimization: 0.30–0.35; perpetration: 0.22–0.29), CR values exceeded 0.60 (victimization: 0.69–0.81; perpetration: 0.67–0.76), indicating acceptable construct validity (Fornell & Larcker, 1981), except for the threats factor (perpetration). HTMT values supported good discriminant validity (victimization: 0.21–0.52; perpetration: 0.26–0.68).

**Table 5.** Reliability, Intercorrelations, Convergent and Discriminant Validity (*N* = 859).

Scale	α	MIIC	CITC	H	MC	SC	T	AVE	CR	HTMT		
										H	MC	SC
Victimization scale	.82	.16	.22–.50									
H	.81	.30	.30–.60	1				0.31	0.81	1		
MC	.72	.27	.32–.53	.20***	1			0.30	0.73	.40	1	
SC	.77	.31	.18–.66	.24***	.19***	1		0.35	0.78	.28	.21	1
T	.66	.29	.34–.52	.30***	.27***	.35***	1	0.33	0.69	.26	.52	.43
Perpetration scale	.82	.17	.13–.53									
H	.77	.27	.37–.54	1				0.25	0.76	1		
MC	.72	.28	.32–.54	.20***	1			0.29	0.69	.43	1	
SC	.71	.30	.08–.71	.20***	.19***	1		0.27	0.67	.51	.26	1
T	.63	.25	.17–.53	.19***	.21***	.26***	1	0.22	0.49	.43	.54	.68

*Note.* H = humiliation; MC = monitoring/control; SC = sexual coercion; T = threats; α = Cronbach’s alpha; MIIC = mean inter-item correlations; CITC = corrected item-total correlations; AVE = average variance extracted; CR = composite reliability; HTMT = heterotrait–monotrait ratio of correlation.

*** *p* < .001, two tailed.

### Prevalence of TFDA

The results support Hypotheses 2a and 2b. As detailed in Table 6, of the 55.8% (*n* = 479) of adolescents involved in TFDA, 11.9% (*n* = 102) reported being victims, 9.4% (*n* = 81) perpetrators, and 34.5% (*n* = 296) victims-perpetrators. Monitoring/control behaviors were the most common (41.2%, *n* = 354) and bidirectional (24.3%, *n* = 209), while sexual coercion (15.9%, *n* = 137) and threats (15.4%, *n* = 132) were the least reported.

**Table 6.** Prevalence of TFDA, Gender, and Age Associations (*N* = 859).

Types of involvement	Total (*N* = 859)	Gender			χ² Fishers’ Exact	Cramers’ *V*	Age Group			χ²	Cramers’ *V*
		Cisgender Boys (*n* = 420)	Cisgender Girls (*n* = 414)	Gender-Diverse (*n* = 25)			12–13 (*n* = 144)	14–16 (*n* = 490)	17–18 (*n* = 225)		
Total					8.29^a^	0.07				13.12^a^,*	0.09
Noninvolvement	380 (44.2)	200 (23.3)	171 (19.9)	9 (1)			72 (8.4)	199 (23.2)	109 (12.7)		
Victims	102 (11.9)	53 (6.2)	44 (5.1)	5 (0.6)			11 (1.3)	72 (8.4)	19 (2.2)		
Perpetrators	81 (9.4)	38 (4.4)	40 (4.7)	3 (0.3)			14 (1.6)	42 (4.9)	25 (2.9)		
Victims-perpetrators	296 (34.5)	129 (15)	159 (18.5)	8 (0.9)			47 (5.5)	177 (20.6)	72 (8.4)		
Humiliation					5.47^b^	0.06					
Noninvolvement	660 (76.8)	322 (37.5)	320 (37.3)	18 (2.1)			105 (12.2)	370 (43.1)	185 (21.5)	6.44^a^	0.06
Victims	72 (8.4)	35 (4.1)	34 (4)	3 (0.3)			13 (1.5)	46 (5.4)	13 (1.5)		
Perpetrators	62 (7.2)	26 (3)	35 (4.1)	1 (0.1)			12 (1.4)	38 (4.4)	12 (1.4)		
Victim-perpetrators	65 (7.6)	37 (4.3)	25 (2.9)	3 (0.3)			14 (1.6)	36 (4.2)	15 (1.7)		
Monitoring/control					16.64^a^,**	0.10				6.85^a^	0.06
Noninvolvement	505 (58.8)	267 (31.1)	225 (26.2)	13 (1.5)			93 (10.8)	274 (31.9)	138 (16.1)		
Victims	79 (9.2)	42 (4.9)	32 (3.7)	5 (0.6)			12 (1.4)	50 (5.8)	17 (2.0)		
Perpetrators	66 (7.7)	25 (2.9)	39 (4.5)	2 (0.2)			13 (1.5)	36 (4.2)	17 (2.0)		
Victim-perpetrators	209 (24.3)	86 (10)	118 (13.7)	5 (0.6)			26 (3)	130 (15.1)	53 (6.2)		
Sexual coercion					6.17^b^	0.06				13.11^a^,*	0.09
Noninvolvement	722 (84.1)	360 (41.9)	341 (39.7)	21(2.4)			128 (14.9)	402 (46.8)	192 (22.4)		
Victims	52 (6.1)	22 (2.6)	30 (3.5)	0 (0)			4 (0.5)	40 (4.7)	8 (0.9)		
Perpetrators	37 (4.3)	19 (2.2)	16 (1.9)	2 (0.2)			3 (0.3)	20 (2.3)	14 (1.6)		
Victim-perpetrators	48 (5.6)	19 (2.2)	27 (3.1)	2 (0.2)			9 (1.0)	28 (3.3)	11 (1.3)		
Threats					8.49^b^	0.07				9.81^a^	0.08
Noninvolvement	727 (84.6)	366 (42.6)	339 (39.5)	22 (2.6)			122 (14.2)	408 (47.5)	197 (22.9)		
Victims	74 (8.6)	30 (3.5)	43 (5)	1 (0.1)			8 (0.9)	47 (5.5)	19 (2.2)		
Perpetrators	31 (3.6)	15 (1.7)	14 (1.6)	2 (0.2)			8 (0.9)	21 (2.4)	2 (0.2)		
Victim-perpetrators	27 (3.1)	9 (1)	18 (2.1)	0 (0)			6 (0.7)	14 (1.6)	7 (0.8)		

*Note*. TFDA = technology-facilitated dating abuse; χ² = chi-square test.

a Pearson chi-Square.

b Fishers’ exact test.

* *p* < .05. **p < .01, two tailed.

### Gender and Age Associations

Hypothesis 3a was not supported, as no significant gender associations were found in the sexual forms. However, a significant association between gender and monitoring/control was found (χ²[6] = 16.64, *p* = .01, Cramer’s *V* = 0.10), with adjusted standardized residuals indicating that cisgender boys were significantly more likely than expected to be uninvolved (*z* = 2.8). In contrast, cisgender girls were more likely than expected to be involved as victims-perpetrators (*z* = 2.7). Notably, the small size of the gender-diverse subsample resulted in small cell counts in some contingency tables, which may have limited the statistical power for group comparisons. To assess the robustness of our findings, we repeated these analyses, excluding non-cisgender participants; the results remained consistent in both direction and statistical significance (see Supplemental Table 1). Hypothesis 3b was partially supported. Significant associations were found between age and overall involvement in TFDA (χ²[6] = 13.12, *p* = .04, Cramer’s *V* = 0.09) and specifically in sexual coercion (χ²[6] = 13.11, *p* = .04, Cramer’s *V* = 0.09), with adjusted standardized residuals indicating that adolescents aged 14 to 16 were significantly more likely than expected to be victims of TFDA (z = 2.9) and sexual coercion (z = 3.0). However, no significant differences were found for the 17 to 18 age group or perpetration.

### Associations with Self-Esteem, HRQoL, and In-Person Dating Violence

The results support Hypotheses 4a, 4b, and 4c. As shown in Table 7, TFDA was negatively correlated with self-esteem (victimization: *r*s= −.15, perpetration: *rs*= −.13), supporting Hypothesis 4a. Similarly, TFDA was negatively correlated with HRQoL (victimization: *r*s= −.07 to −.20, perpetration: *rs* = −.10 to −.20), supporting Hypothesis 4b. Furthermore, TFDA victimization correlated positively with in-person dating victimization (*r*s= .17–.40), while TFDA perpetration correlated positively with in-person dating perpetration (*rs*= .10–.36), supporting Hypothesis 4c.

**Table 7.** Correlations Between TFDA, Self-Esteem, HRQoL, and In-Person Dating Violence.

Variables	Victimization Scale					Perpetration Scale				
	H	MC	SC	T	Total	H	MC	SC	T	Total
Self-esteem	−.03	−.15***	−.10**	−.09**	−.15***	−.05	−.15***	−.02	−.03	−.13***
HRQoL										
Physical well-being	−.05	−.04	−.07*	−.12***	−.07*	−.03	−.15***	−.02	−.08*	−.14***
Psychological well-being	−.09**	−.19***	−.12***	−.15***	−.20***	−.09**	−.19***	−.05	−.04	−.19***
Parent relations	−.08*	−.15***	−.18***	−.12***	−.18***	−.14***	−.16***	−.08*	−.05	−.16***
Peers and social support	−.06	−.10**	−.11**	−.07	−.14***	−.08*	−.10**	−.08*	−.03	−.10**
School environment	−.08*	−.12***	−.06	−.11**	−.13***	−.08*	−.16***	−.01	−.03	−.14***
Total	−.09**	−.18***	−.15***	−.14***	−.20***	−.11**	−.21***	−.06	−.05	−.20***
In-person dating violence										
Physical abuse	.26***	.07	.17***	.12**	.17***	.19***	.08	.04	.16***	.10*
Emotional abuse	.21***	.33***	.17***	.33***	.36***	.18***	.35***	.09	.12**	.32***
Relational abuse	.26***	.24***	.16***	.30***	.27***	.25***	.11*	.15**	.23***	.21***
Sexual abuse	.15**	.13**	.34***	.21***	.24***	.26***	.09	.19***	.17***	.19***
Threats	.21***	.12**	.19***	.26***	.20***	.08	.10*	.09	.14***	.11*
Total	.25***	.35***	.24***	.36***	.40***	.25***	.35***	.13**	.18***	.36***

*Note.* The victimization scale was correlated with in-person dating victimization, while the perpetration scale was correlated with in-person dating perpetration. TFDA = technology-facilitated dating abuse; H = humiliation; MC = monitoring/control; SC = sexual coercion; T = threats; self-esteem (*n* = 769); HRQoL = health-related quality of life (*n* = 769); in-person dating violence (*n* = 426).

* *p* < .05. **p < .01. ***p < .001, two tailed.

## Discussion

The main aims of this pioneering study were to evaluate the utility of the TAR scale among Portuguese adolescents, estimate prevalence rates of TFDA, and examine its associations with gender, age, self-esteem, HRQoL, and in-person dating violence.

Hypothesis 1 proposed that the Portuguese version of the TAR scale would confirm its original four-factor structure with good psychometric properties among Portuguese adolescents. The results supported this hypothesis, confirming the validity of the original four-factor structure in the Portuguese context: *Humiliation* (ridicule, devaluation, embarrassment), *Monitoring/Control* (privacy intrusion, control over activities and interactions), *Sexual Coercion* (sexual pressure, image-based sexual abuse), and *Threats* (intimidation, expressed intent to harm; Brown & Hegarty, 2021). The victimization model showed a good fit without requiring modifications. In contrast, the perpetration model required limited error correlations between items with similar wording, content, or latent factors—decisions guided by theory and supported by modification indices (Marôco, 2021). Although Items 25 (victimization), 24, and 28 (perpetration) presented lower factor loadings, they were retained due to their theoretical, methodological, and clinical relevance. These items capture unique and emerging forms of TFDA—such as image-based sexual abuse—and address a gap in available validated measures for assessing these specific behaviors (e.g., Rocha-Silva et al., 2021), thereby contributing to a more comprehensive assessment of this phenomenon. While their lower endorsement may reflect either low prevalence or (un)intentional underreporting (e.g., Ellyson et al., 2021; Reed et al., 2017; Stonard, 2021; Zweig et al., 2013), victims have described some of these behaviors as among the most harmful TFDA experiences (e.g., Reed et al., 2017), highlighting their clinical importance for screening and intervention. Additionally, we preserved structural symmetry between the victimization and perpetration subscales to enable meaningful comparisons between roles, particularly since dual involvement is the most reported pattern in adolescent populations (e.g., Ellyson et al., 2021). Notably, none of these items exhibited low factor loadings across both subscales, further supporting their retention without compromising the model’s overall validity. Cronbach’s alpha for the Threats factor was slightly below .70, but values above .60 are acceptable in exploratory research (Hair et al., 2019), especially with MIIC values within recommended ranges. Although items 24, 25, and 28 had CITC values below thresholds (Nunnally & Bernstein, 1994), their removal did not significantly impact reliability, further supporting their retention. Convergent validity showed AVE values below 0.50, but CR exceeded 0.60 for most factors, meeting Fornell and Larcker’s (1981) criteria, except for the Threats factor in the perpetration scale, likely due to the lower prevalence and reduced loading of item 28. Discriminant validity was supported, confirming the scale’s overall robustness (Henseler et al., 2015).

Consistent with Hypothesis 2a, TFDA was prevalent among adolescents (55.8%, *n* = 479), with a large proportion involved as both victims and perpetrators (34.5%, *n* = 296). Overall, these results align with international studies (e.g., Ellyson et al., 2021; Stonard, 2021; Zweig et al., 2013) but differ from those of the Portuguese study conducted by UMAR (2025), which assessed only victimization using a limited number of items and reported a prevalence of 19.8% among adolescents. These differences likely reflect the TAR scale’s broader coverage of abuse typologies and its parallel assessment of both victimization and perpetration roles. Several factors may explain these patterns. Developmentally, adolescents are undergoing brain development and physiological changes (Spear, 2000), along with identity exploration (Erikson, 1963). During this stage, they may lack key interpersonal skills, such as communication, negotiation, and the ability to anticipate moral, social, and legal consequences (e.g., Selman et al., 1986)—and may struggle to recognize unhealthy dating behaviors (e.g., Baker & Carreño, 2016; Lucero et al., 2014; Stonard et al., 2017). Technology further mediates both the opportunities for connection with a partner and the risk of TFDA (ecological techno-subsystem; Johnson, 2010). In our sample, most adolescents communicated daily with their partner—often for over 5 hr—and frequently used technology to share personal content, engage in sexual intimacy, manage conflict, and reconnect after arguments. Furthermore, the accessibility of these forms of communication and the perceived comfort behind screens can amplify power and control, while reducing accountability and facilitating both proactive and reactive aggression (online disinhibition effect; Suler, 2004). Social contexts—including family, peers, school, and community (Caridade & Braga, 2020), alongside cultural and structural conditions in Portugal, also help shape these patterns. Portugal is a small European country (approximately 10.6 million inhabitants; Instituto Nacional de Estatística [National Institute of Statistics; INE], 2024) that, despite ranking seventh globally for safety (Institute for Economics & Peace, 2024), continues to face notable economic, educational, and gender inequalities. For example, 11.3% of children up to 15 years old live in material and social deprivation (INE, 2025), the rate of low educational attainment among adults aged 25 to 64 is among the highest in the OECD (Organisation for Economic Co-operation and Development, 2024), and the country scores below the EU average on gender equality (European Institute for Gender Inequality, 2024). Nonetheless, recent years have seen significant legislative and policy developments aimed at improving recognition of TFDA, protecting victims, and supporting the rehabilitation of perpetrators. In 2013, the Portuguese Penal Code was amended to expand the definition of domestic violence to include dating relationships. In 2018, an aggravating circumstance was introduced for the nonconsensual online dissemination of a victim’s private data. National strategies—such as the Child Guarantee Action Plan (2022–2030) and the Strategy for the Rights of Children and Young People (2025–2035)—alongside school-based campaigns by organizations such as the APAV and the Public Security Police, have prioritized the promotion of a culture of nonviolence and digital safety. The key recommendations continue to call for the integration of education on unhealthy dating behaviors, assertive communication, emotional regulation, critical thinking, conflict resolution, empathy, intervention skills, digital literacy, and anti-TFDA campaigns into adolescents’ formal and informal contexts. They also stress the ongoing need to sensitize caregivers, school-based services, and formal support systems to address the needs of victims and perpetrators.

Consistent with Hypothesis 2b, monitoring/control behaviors emerged as the most common (41.2%, *n* = 354) and bidirectional (24.3%, *n* = 209) form of TFDA. Qualitative research indicates that adolescents often perceive these behaviors as expressions of love, care, trust, and commitment, with practices like sharing account passwords being common early in relationships (e.g., Baker & Carreño, 2016; Lucero et al., 2014; Stonard et al., 2017). However, such behaviors are also associated with jealousy and are often dismissed as problematic unless perceived as extreme (e.g., Baker & Carreño, 2016; Lucero et al., 2014), which may reinforce victims-perpetrator overlap.

Hypothesis 3a expected that cisgender girls would more often be victims and cisgender boy perpetrators of sexual forms of TFDA. This was not supported, as no significant gender differences were found in the sexual forms. However, cisgender boys were more often uninvolved in monitoring/control behaviors, while cisgender girls were more frequently involved as victims—perpetrators. Traditional gender norms may further explain these patterns. Heteronormative masculinity—emphasizing dominance, self-reliance, and independence—may discourage cisgender boys from disclosing their experiences. In contrast, cisgender girls are more likely to discuss such behaviors (both their own and their partners’; e.g., Baker & Carreño, 2016), viewing them as necessary for maintaining relationships (e.g., Lucero et al., 2014) or attributing them to protective or caring traits (e.g., Stonard et al., 2017) associated with heteronormative femininity. Addressing these issues requires deconstructing romantic myths to help adolescents recognize abuse, seek support, or end unhealthy relationships; creating safe, stigma-free spaces for victims to share their experiences; and further examining the role of gender stereotypes and socialization practices in TFDA.

Hypothesis 3b proposed that middle to late adolescents (aged 14–18) would be more frequently involved in both victimization and perpetration of sexual and nonsexual forms of TFDA. This hypothesis was partially supported, with adolescents aged 14 to 16 reporting higher victimization, particularly in sexual coercion. This vulnerability may stem from greater involvement in dating and sexual relationships (e.g., Connolly et al., 2014; Thulin et al., 2022), engagement in risk-taking behaviors such as using the Internet to flirt, initiate relationships, engage in sexting, and consume pornography (e.g., Lenhart et al., 2015; Widman et al., 2021), reduced parental supervision, and/or decreased influence from adults/parents (e.g., Vale et al., 2018; Thulin et al., 2022). Early identification of vulnerable subpopulations and age-appropriate prevention strategies are crucial to prevent TFDA from becoming entrenched in adolescence and persisting into adulthood.

TFDA was negatively correlated with self-esteem (H4a) and HRQoL (H4b) and positively correlated with in-person dating violence (H4c), aligning with previous studies (e.g., Ortega-Barón et al., 2022; Rodríguez-deArriba et al., 2024; Smith et al., 2018). These associations suggest that TFDA may contribute to reduced self-esteem and HRQoL. However, it is also plausible that adolescents with lower self-esteem and HRQoL are more vulnerable to TFDA involvement. This underscores that TFDA has causes and consequences, likely impacting multiple aspects of adolescents’ lives (Caridade & Braga, 2020). Additionally, TFDA may precede, coexist with, or result from in-person dating violence (e.g., Rodríguez-deArriba et al., 2024), highlighting their interconnectedness and the potential for a cycle where one form of abuse reinforces the other. While TFDA shares similarities with in-person dating violence, its technological nature introduces unique challenges—such as constant accessibility that allows perpetrators to exert power and control beyond temporal and spatial boundaries, evolving abusive methods, broad audience reach, and the permanence of digital evidence—which may further hinder recovery (Afrouz & Vassos, 2024; Hellevik, 2019; Vale et al., 2020). Longitudinal studies are needed to determine these relationships’ directionality and examine potential mediating factors, such as coping, help-seeking behaviors, and resilience.

### Limitations and Future Research

Some limitations should be noted, offering directions for future research: (a) the cross-sectional design limits causal inferences; (b) the convenience sample restricts the generalizability of the findings to adolescents in other Portuguese regions; (c) although the sample included both cisgender and gender-diverse adolescents, small subgroup sizes may have limited the statistical power for detailed comparisons. Future diversity-focused research should include larger and more diverse samples to examine how TFDA may vary across gender identity, ethnicity, sexual orientation, socioeconomic status, and rural versus urban contexts; (d) as only adolescents present on the day of data collection with signed parental consent were eligible, there is potential for nonresponse bias (e.g., school absenteeism may correlate with psychosocial risk factors relevant to TFDA, which could affect prevalence estimates; (e) self-reports may be affected by recall errors and social desirability bias; (f) adolescents were asked about specific abusive behaviors, rather than self-identifying as victims and/or perpetrators; (g) limiting TFDA assessment to the past 12 months may have underestimated lifetime prevalence; (h) the Portuguese version of the TAR scale used binary phrasing (“He/She”) for victimization items and “I” for perpetration, which limits inclusivity and highlights the importance of adopting more gender-inclusive language in future scale adaptations; (i) the number of relationships and dating partners was not assessed, so the reported prevalence may reflect incidents occurring within one or across multiple relationships. As such, the observed 34.5% overlap between victimization and perpetration may reflect either mutual abuse within a single relationship and/or role fluidity across different relationships; (j) future research should prioritize measurement invariance across gender and age; (k) and, while this study provides important insights into TFDA among Portuguese adolescents, the findings may not fully generalize to adolescents in other countries with different patterns of digital media use, attitudes and norms surrounding gender roles, dating relationships, and dating violence, as well as differing legal frameworks, prevention initiatives, and support services for victims and perpetrators.

## Conclusion

The TAR scale effectively captures TFDA, addressing the needs of adolescents, researchers, and practitioners. Internationally, its validation facilitates cross-country comparative studies, while in Portugal, it marks significant progress and lays the groundwork for future advancements by: (a) enhancing the knowledge related to prevalence rates, etiology, risk and protective factors, and consequences; (b) enabling early identification and referrals of victims and perpetrators to appropriate services; (c) supporting the development of evidence-based prevention and intervention strategies for victims, perpetrators, and allies/bystanders; (d) guiding the allocation of funding, resources, and support for services working with adolescents; and (e) increasing public awareness. It also highlights the need for a whole-of-society strategic response, engaging communities—adolescents, parents, teachers, clinicians, child protection professionals, researchers, technology companies, and legal and policy practitioners. Such a collective effort is essential to protect adolescents during this critical developmental stage, foster healthy adult relationships, and reinforce that TFDA is unacceptable and will not be tolerated.

## Supplemental Material

sj-docx-1-jiv-10.1177_08862605251368832 – Supplemental material for Technology-Facilitated Abuse in Relationships Scale: Psychometric Properties of Victimization and Perpetration Among Portuguese Adolescents Supplemental material, sj-docx-1-jiv-10.1177_08862605251368832 for Technology-Facilitated Abuse in Relationships Scale: Psychometric Properties of Victimization and Perpetration Among Portuguese Adolescents by Maria Vale and Marlene Matos in Journal of Interpersonal Violence

sj-docx-2-jiv-10.1177_08862605251368832 – Supplemental material for Technology-Facilitated Abuse in Relationships Scale: Psychometric Properties of Victimization and Perpetration Among Portuguese Adolescents Supplemental material, sj-docx-2-jiv-10.1177_08862605251368832 for Technology-Facilitated Abuse in Relationships Scale: Psychometric Properties of Victimization and Perpetration Among Portuguese Adolescents by Maria Vale and Marlene Matos in Journal of Interpersonal Violence

## Data Availability

The data supporting this study’s findings are available from the corresponding author, [Maria Vale], upon reasonable request.

## Notes

1. Descriptively, the group excluded for dating-related reasons (*n* = 862) was mainly composed of cisgender boys (*n* = 461, 53.5%), aged 14 to 16 (*n* = 465, 53.9%), White (*n* = 793, 92%), heterosexual (*n* = 741, 86%), attending public schools (n = 688, 79.8%), enrolled in grades 7 to 8 (n = 350, 40.6%), and living in rural areas (n = 435, 50.5%). This profile is broadly like the included group, although the latter was slightly more likely to be enrolled in higher school grades (9–10) and to reside in urban areas.

2. In this manuscript, cisgender refers to individuals whose gender identity aligns with their sex assigned at birth; binary trans refers to individuals identifying as boys or girls; non-binary refers to individuals identifying outside the gender binary; and gender-questioning refers to individuals who are questioning their gender identity. These categories are understood as fluid rather than fixed.
