# Supplementary material for: Technology-Facilitated Abuse in Relationships Scale: Psychometric Properties of Victimization and Perpetration Among Portuguese Adolescents
Source: J Interpers Violence. 2025 Oct 1;41(21-22):8912–43. doi: 10.1177/08862605251368832 (PMC13669468; doi:10.1177/08862605251368832)
Supplement: Supplementary file 1 [file sj-docx-1-jiv-10.1177_08862605251368832.docx]

**Supplemental Figure 1**

*CFA model with* *standardized factor loadings for the TAR victimization scale (left) and TAR perpetration scale (right)*
