# Supplementary material for: Technology-Facilitated Abuse in Relationships Scale: Psychometric Properties of Victimization and Perpetration Among Portuguese Adolescents
Source: J Interpers Violence. 2025 Oct 1;41(21-22):8912–43. doi: 10.1177/08862605251368832 (PMC13669468; doi:10.1177/08862605251368832)
Supplement: Supplementary file 2 [file sj-docx-2-jiv-10.1177_08862605251368832.docx]

| **Supplemental Table 1.**  *Gender differences in TFDA (N = 834)* | | | |  |  |
| --- | --- | --- | --- | --- | --- |
|  | Total  (*N* = 834) | Cisgender  boys  (*n* = 420) | Cisgender girls  (*n* = 414) | χ² | Cramer’s V |
| Total |  |  |  | 6.24 | 0.09 |
| Non-involvement | 371 (44.5) | 200 (24) | 171 (20.5) |  |  |
| Victims | 97 (11.6) | 53 (6.4) | 44 (5.3) |  |  |
| Perpetrators | 78 (9.4) | 38 (4.6) | 40 (4.8) |  |  |
| Victim-Perpetrators | 288 (34.5) | 129 (15.5) | 159 (19.1) |  |  |
| Humiliation |  |  |  | 3.63 | 0.07 |
| Non-involvement | 642 (77) | 322 (38.6) | 320 (38.4) |  |  |
| Victims | 69 (8.3) | 35 (4.2) | 34 (4.1) |  |  |
| Perpetrators | 61 (7.3) | 26 (3.1) | 35 (4.2) |  |  |
| Victim-Perpetrators | 62 (7.4) | 37 (4.4) | 25 (3) |  |  |
| Monitoring/control |  |  |  | 12.98** | 0.13 |
| Non-involvement | 492 (59) | 267 (32) | 225 (27) |  |  |
| Victims | 74 (8.9) | 42 (5) | 32 (3.8) |  |  |
| Perpetrators | 64 (7.7) | 25 (3) | 39 (4.7) |  |  |
| Victim-Perpetrators | 204 (24.5) | 86 (10.3) | 118 (14.1) |  |  |
| Sexual coercion |  |  |  | 3.35 | 0.06 |
| Non-involvement | 701 (84.1) | 360 (43.2) | 341 (40.9) |  |  |
| Victims | 52 (6.2) | 22 (2.6) | 30 (3.6) |  |  |
| Perpetrators | 35 (4.2) | 19 (2.3) | 16 (1.9) |  |  |
| Victim-Perpetrators | 46 (5.5) | 19 (2.3) | 27 (3.2) |  |  |
| Threats |  |  |  | 6.34 | 0.09 |
| Non-involvement | 705 (84.5) | 366 (43.9) | 339 (40.6) |  |  |
| Victims | 73 (8.8) | 30 (3.6) | 43 (5.2) |  |  |
| Perpetrators | 29 (3.5) | 15 (1.8) | 14 (1.7) |  |  |
| Victim-Perpetrators | 27 (3.2) | 9 (1.1) | 18 (2.2) |  |  |
| *Note*. TFDA = Technology-facilitated dating abuse; χ² = Chi-square test; ***p* < .01, two tailed. | | | | | |
